# Supplementary material for: Inflammation and IL-4 regulate Parkinson’s and Crohn’s disease associated kinase LRRK2
Source: EMBO Rep. 2025 May 20;26(13):3327–56. doi: 10.1038/s44319-025-00473-x (PMC12238514; doi:10.1038/s44319-025-00473-x)
Supplement: Supplementary file 1 — Appendix [file 44319_2025_473_MOESM1_ESM.pdf]

Dikovskaya et al. Inflammation and IL-4 regulate Parkinson’s and Crohn’s disease associated kinase LRRK2.

Appendix

Table of context:

Appendix Figure S1. Gating strategy for splenocytes and lamina propria cells. …... page 2

Appendix Figure S2. Analysis of Lrrk2 pathway in kidneys (A) and brain (B) of EGFP-LRRK2-KI mouse. …………………………………………………………………………..page 3

Appendix Figure S3. Gating strategy for mouse splenic B-cell subtypes. ……......... page 4

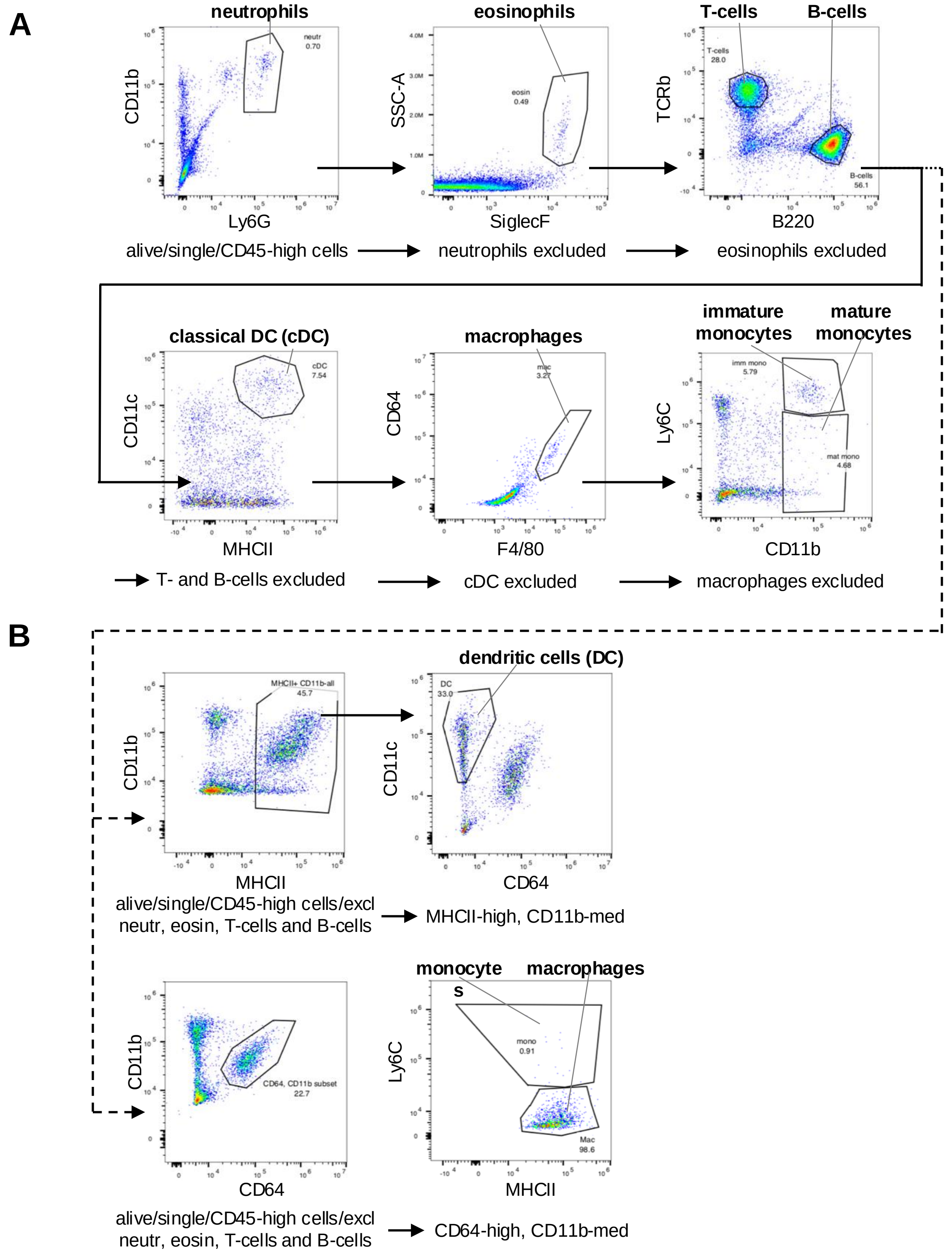

Appendix Figure S1. Gating strategy for splenocytes and lamina propria cells. **A.** Splenocytes gating. Immune cells were gated from live, single CD45+ cells followed by sequential identification and exclusion of: neutrophils (CD11b^{+}/Ly6G^{hi}); eosinophils (SiglecF^{+}/ SSC-high); T- cells (TCRb+) and B-cells (B220-high); classical dendritic cells (cDC, CD11b^{hi}/MHCII^{hi}); macrophages (CD64+/F4/80+) and immature and mature monocytes (CD11b^{hi}/Ly6C^{hi} and CD11b^{hi}/Ly6C^{lo} respectively). **B.** Lamina propria cells gating. The staining and initial gating was identical to A up to the exclusion of T - and B-cells, after which DCs were defined as CD11b$^{+/-}$/MHCII^{hi} then gating on CD11c^{hi}/CD64^{-} cells. Macrophages and monocytes were identified as CD64+/ CD11^{int} cells, then Ly6C^{+} for monocytes and for Ly6C^{-}/MHCII^{+} for macrophages.

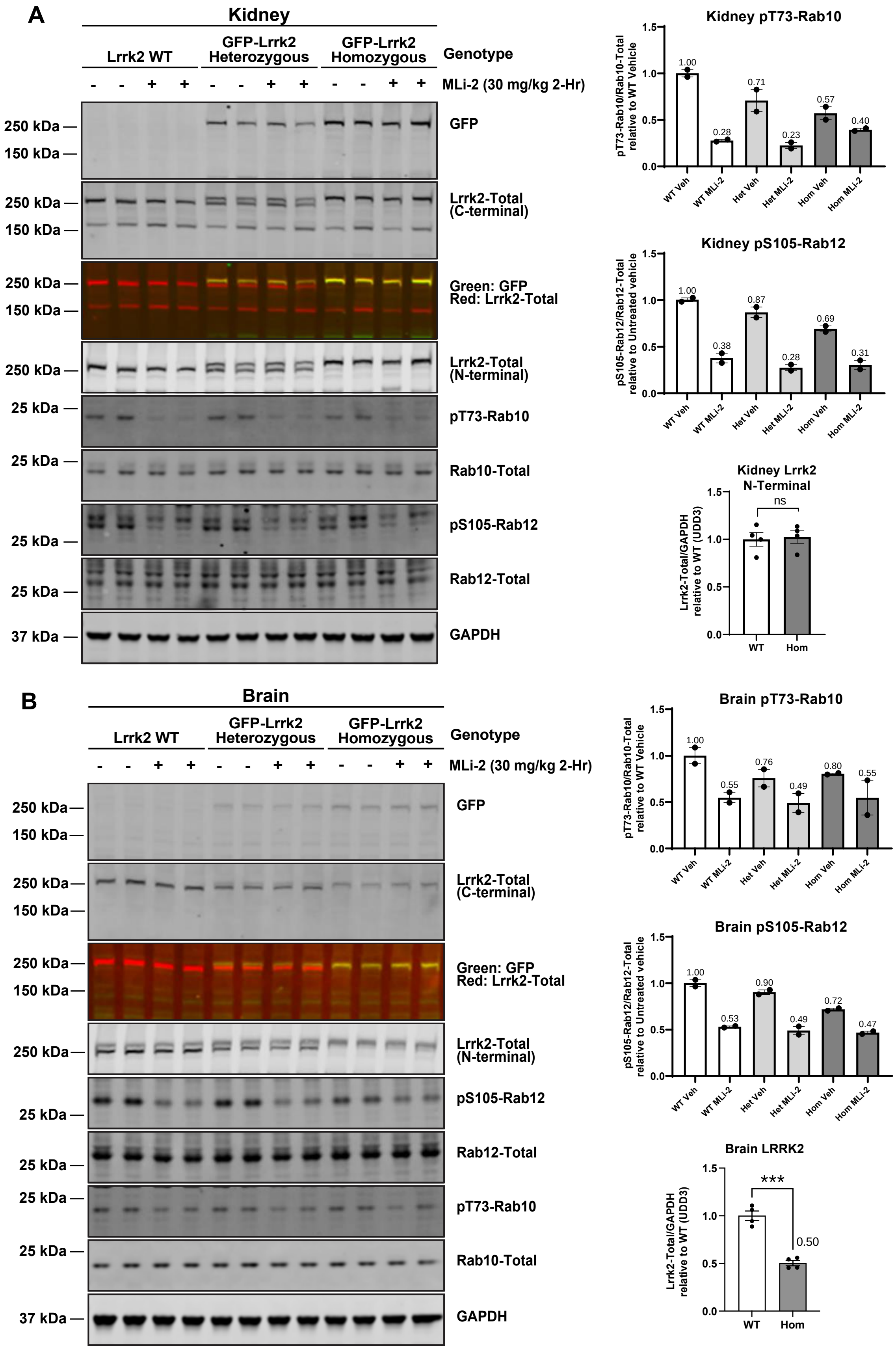

Appendix Figure S2. Analysis of Lrrk2 pathway in kidneys (A) and brain (B) of EGFP-LRRK2-KI mouse. 3-month-old WT, Heterozygous and Homozygous EGFP-LRRK2-KI mice were treated with or without 30 mg/kg MLi-2 for 2h prior to tissue collection and processed as in Fig. EV4. Quantification of Lrrk2-substrate phosphorylation of pT73-Rab10 and pS105-Rab12 relative to total levels (n = 2), and total Lrrk2 levels relative to GAPDH for both N-terminal antibodies (n = 4) are shown as mean ± SEM for each tissue. Statistical significance calculated by unpaired 2-tailed T-test, with the values *** - p = 0.0003 or "ns" - nonsignificant. Each lane indicated sample derived from a different mouse tissue.

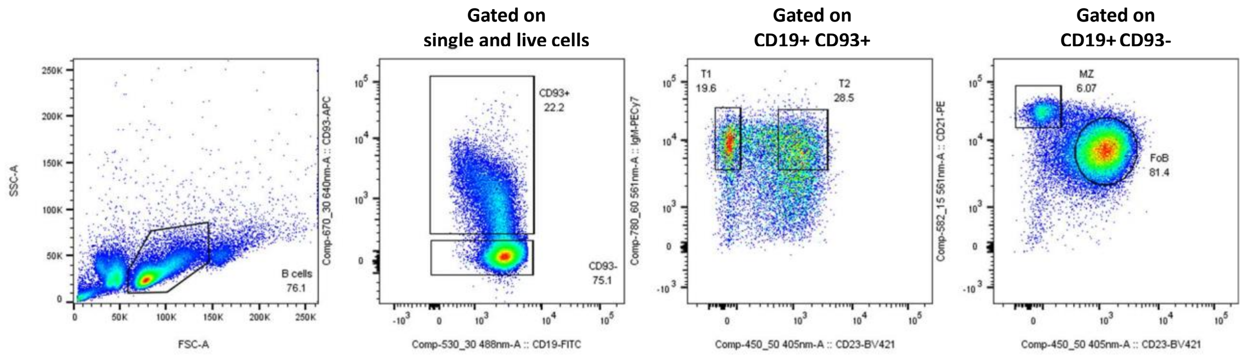

Appendix Figure S3. Gating strategy for mouse splenic B-cell subtypes. Splenic B-cells were isolated using pan B-cell isolation kit by negative selection and stained with DAPI and antibodies for surface markers. Live single CD19^{+} B-cells were digitally separated into CD93^{hi}/CD23^{-} T1 transitional B-cells and CD93^{hi}/CD23^{hi} T2 transitional B-cells, and CD93^{-} mature B-cells were further subdivided CD23^{hi}/CD21^{+} follicular B-cells (Fo) and CD23^{-}/CD21^{hi} marginal zone B-cells (MZ).
