# Supplementary material for: Inflammation and IL-4 regulate Parkinson’s and Crohn’s disease associated kinase LRRK2
Source: EMBO Rep. 2025 May 20;26(13):3327–56. doi: 10.1038/s44319-025-00473-x (PMC12238514; doi:10.1038/s44319-025-00473-x)
Supplement: Supplementary file 3 — Source data Fig. 1 [file 44319_2025_473_MOESM3_ESM.zip › Figure 1/1A/EMBOR-2024-60209V1-T-SourceDataForFigureFigure1A.pptx]

## Slide 1
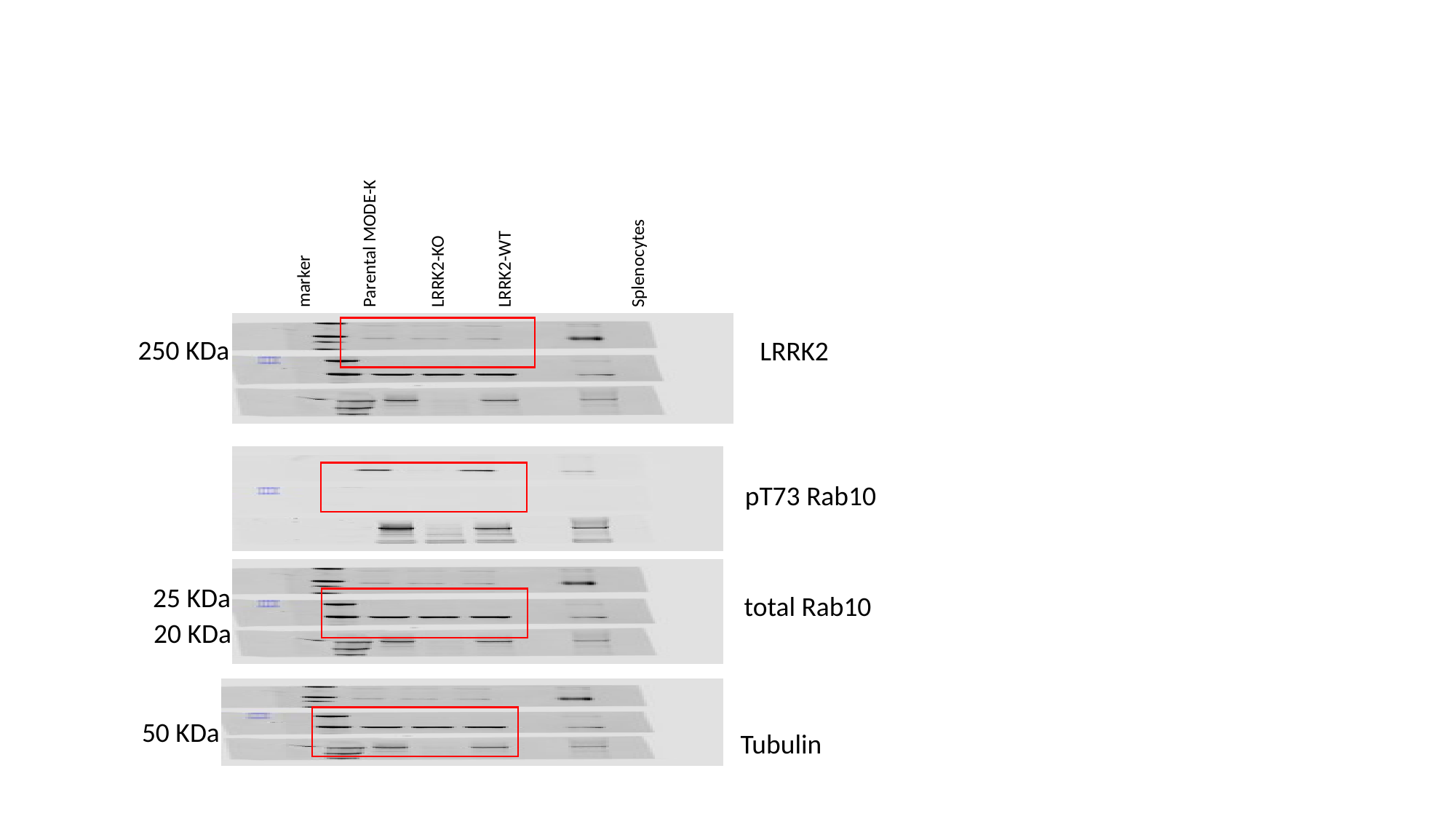

marker
Parental MODE-K
LRRK2-WT
LRRK2-KO
Splenocytes
250 KDa
LRRK2
pT73 Rab10
25 KDa
total Rab10
20 KDa
50 KDa
Tubulin
