# Supplementary material for: Inflammation and IL-4 regulate Parkinson’s and Crohn’s disease associated kinase LRRK2
Source: EMBO Rep. 2025 May 20;26(13):3327–56. doi: 10.1038/s44319-025-00473-x (PMC12238514; doi:10.1038/s44319-025-00473-x)
Supplement: Supplementary file 3 — Source data Fig. 1 [file 44319_2025_473_MOESM3_ESM.zip › Figure 1/1C/EMBOR-2024-60209V1-T-SourceDataForFigureFigure1C.pptx]

## Slide 1
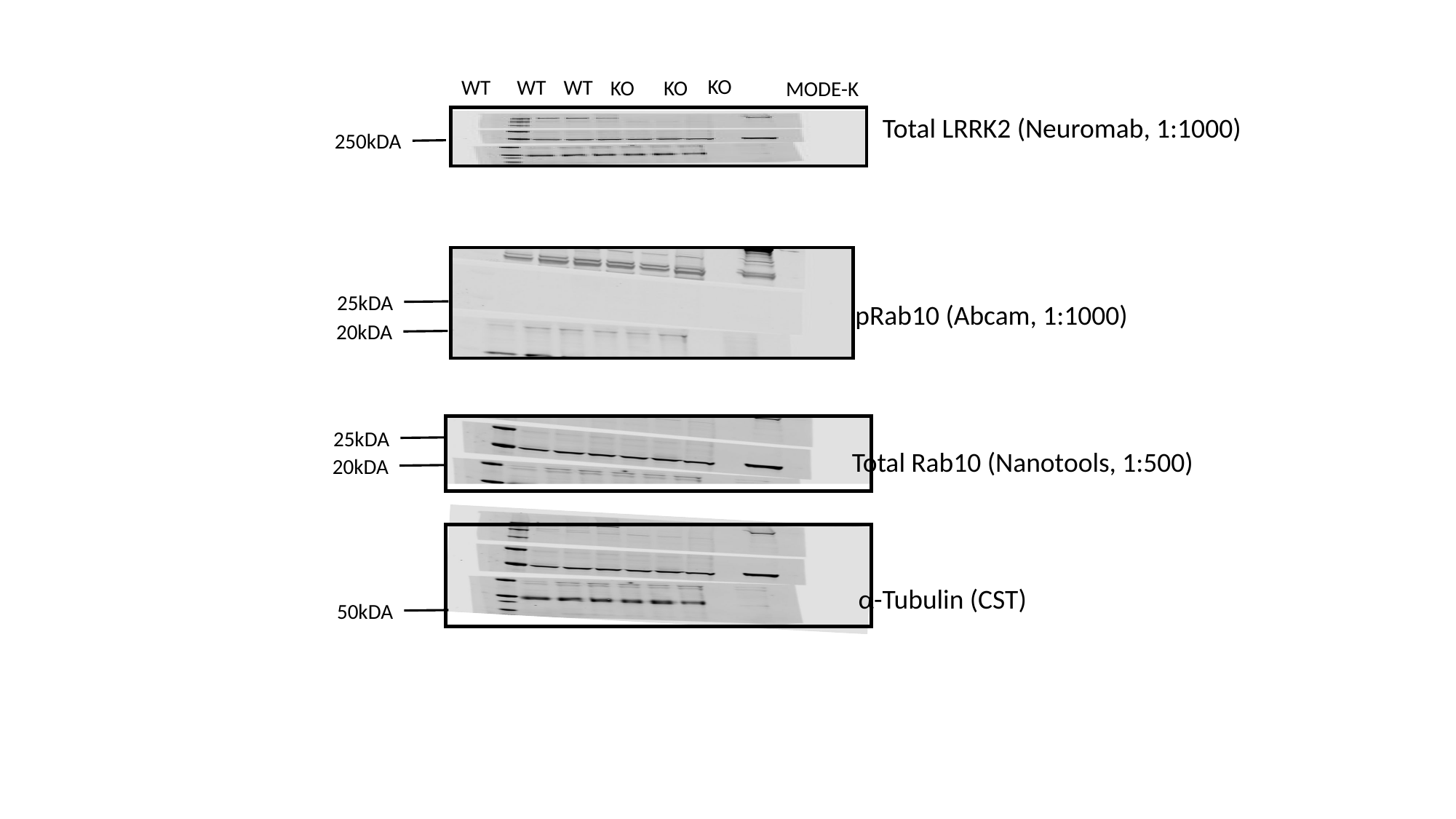

KO
WT
WT
WT
KO
KO
MODE-K
Total LRRK2 (Neuromab, 1:1000)
250kDA
25kDA
pRab10 (Abcam, 1:1000)
20kDA
25kDA
Total Rab10 (Nanotools, 1:500)
20kDA
α-Tubulin (CST)
50kDA

## Slide 2
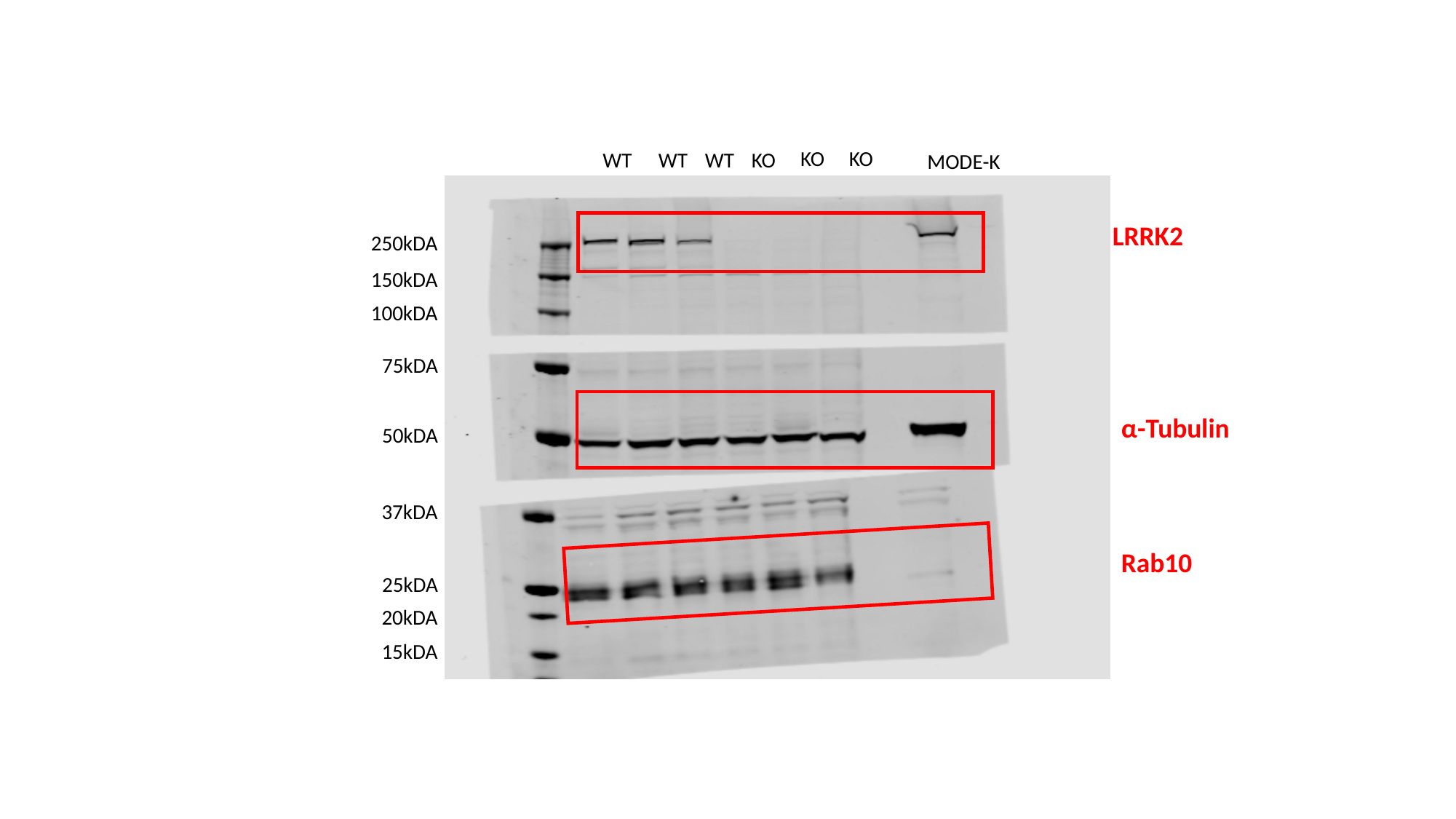

KO
KO
WT
WT
WT
KO
MODE-K
LRRK2
250kDA
150kDA
100kDA
75kDA
α-Tubulin
50kDA
37kDA
Rab10
25kDA
20kDA
15kDA

## Slide 3
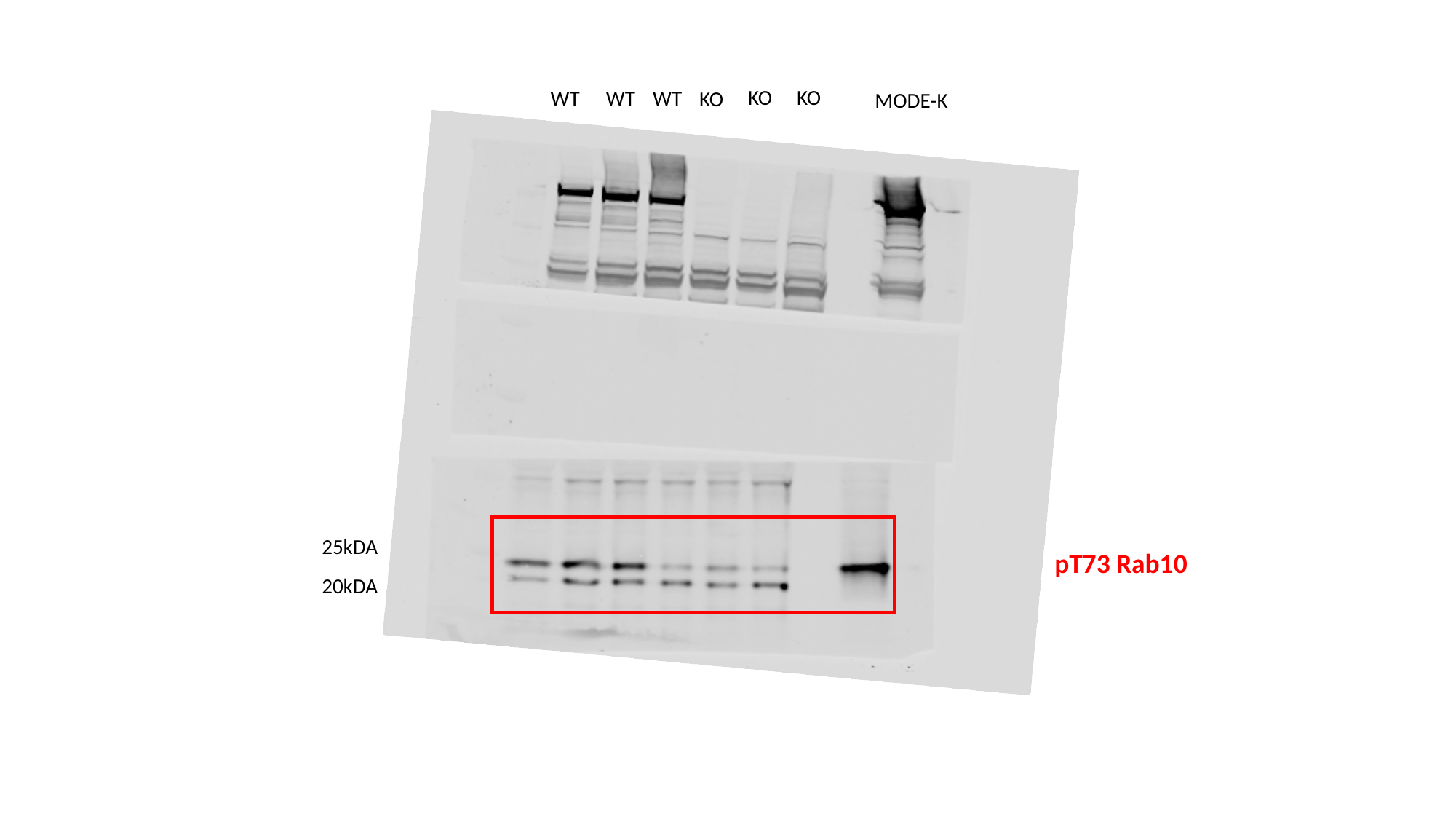

KO
KO
WT
WT
WT
KO
MODE-K
25kDA
pT73 Rab10
20kDA
