# Supplementary material for: Inflammation and IL-4 regulate Parkinson’s and Crohn’s disease associated kinase LRRK2
Source: EMBO Rep. 2025 May 20;26(13):3327–56. doi: 10.1038/s44319-025-00473-x (PMC12238514; doi:10.1038/s44319-025-00473-x)
Supplement: Supplementary file 5 — Source data Fig. 3 [file 44319_2025_473_MOESM5_ESM.zip › Figure 3/3C/EMBOR-2024-60209V2-T-SourceDataForFigureFigure3C_blots.pptx]

## Slide 1
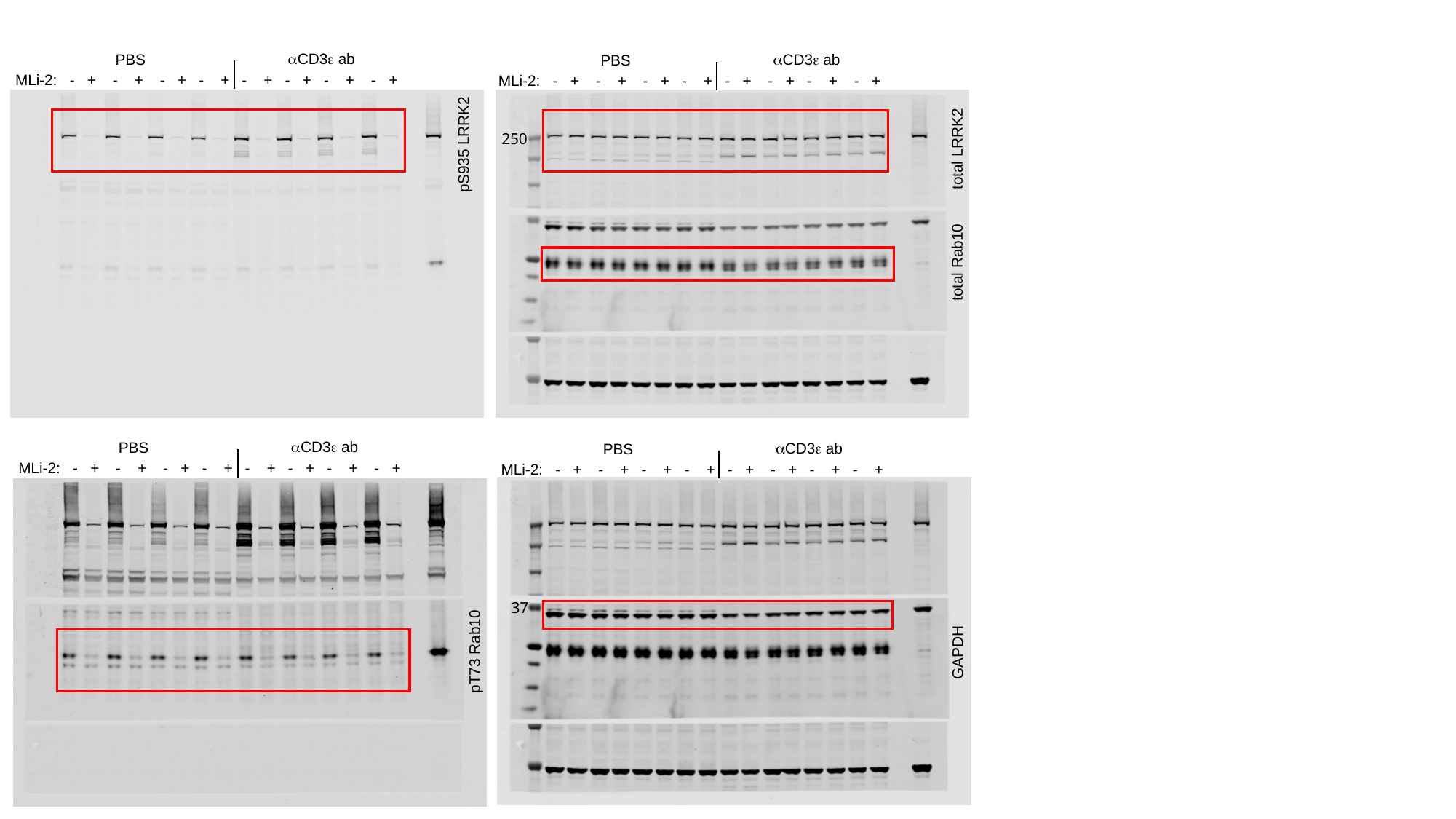

aCD3e ab
PBS
aCD3e ab
PBS
MLi-2: - + - + - + - + - + - + - + - +
MLi-2: - + - + - + - + - + - + - + - +
250
pS935 LRRK2
total LRRK2
total Rab10
aCD3e ab
PBS
aCD3e ab
PBS
MLi-2: - + - + - + - + - + - + - + - +
MLi-2: - + - + - + - + - + - + - + - +
37
pT73 Rab10
GAPDH
