# Supplementary material for: Inflammation and IL-4 regulate Parkinson’s and Crohn’s disease associated kinase LRRK2
Source: EMBO Rep. 2025 May 20;26(13):3327–56. doi: 10.1038/s44319-025-00473-x (PMC12238514; doi:10.1038/s44319-025-00473-x)
Supplement: Supplementary file 7 — Source data Fig. 5 [file 44319_2025_473_MOESM7_ESM.zip › Figure 5/5G/EMBOR-2024-60209V1-T-SourceDataForFigureFigure5G_Blots.pptx]

## Slide 1
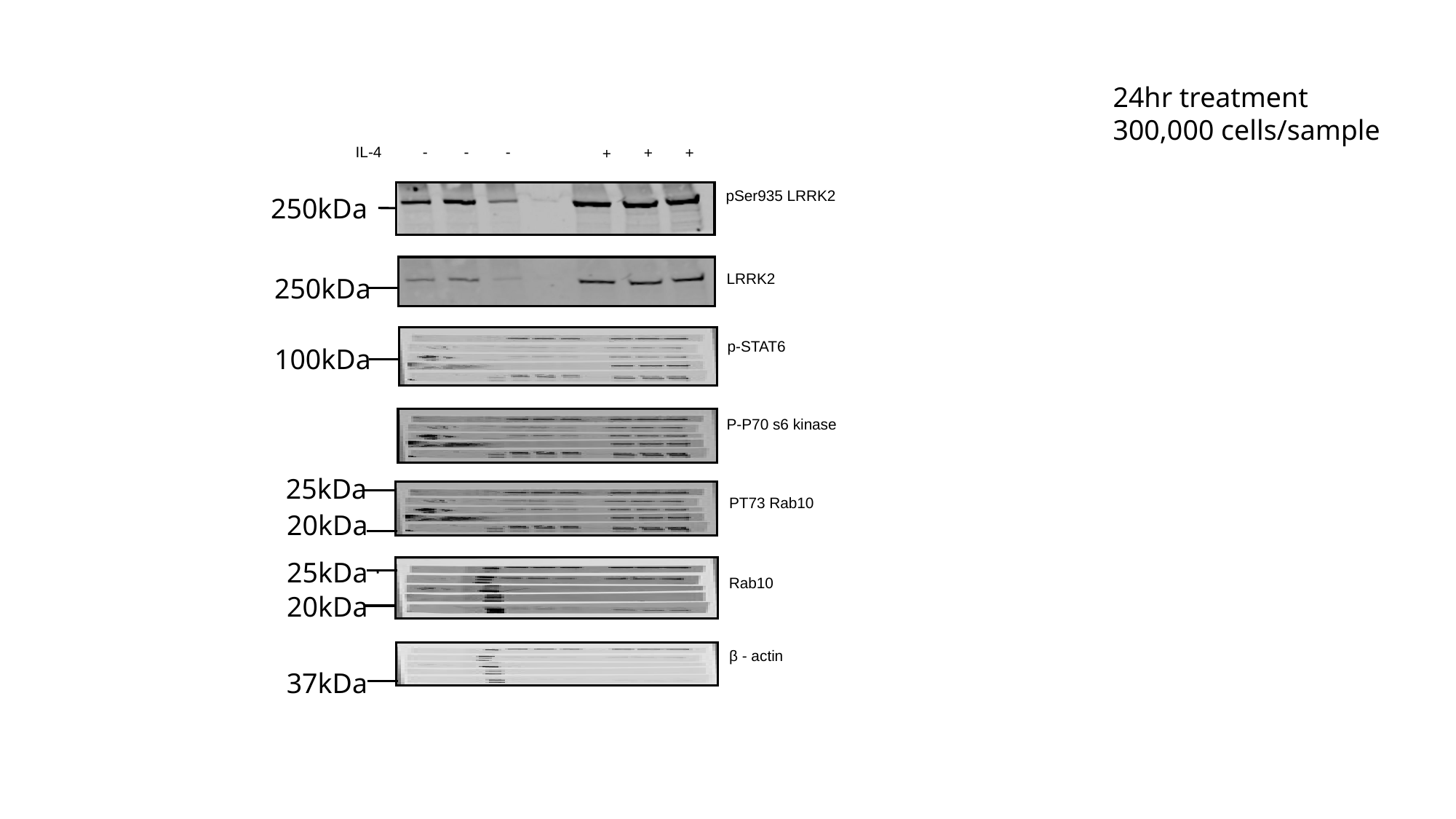

24hr treatment
300,000 cells/sample
-
-
IL-4
-
+
+
+
pSer935 LRRK2
250kDa
LRRK2
250kDa
p-STAT6
100kDa
P-P70 s6 kinase
25kDa
PT73 Rab10
20kDa
25kDa
Rab10
20kDa
β - actin
37kDa

## Slide 2
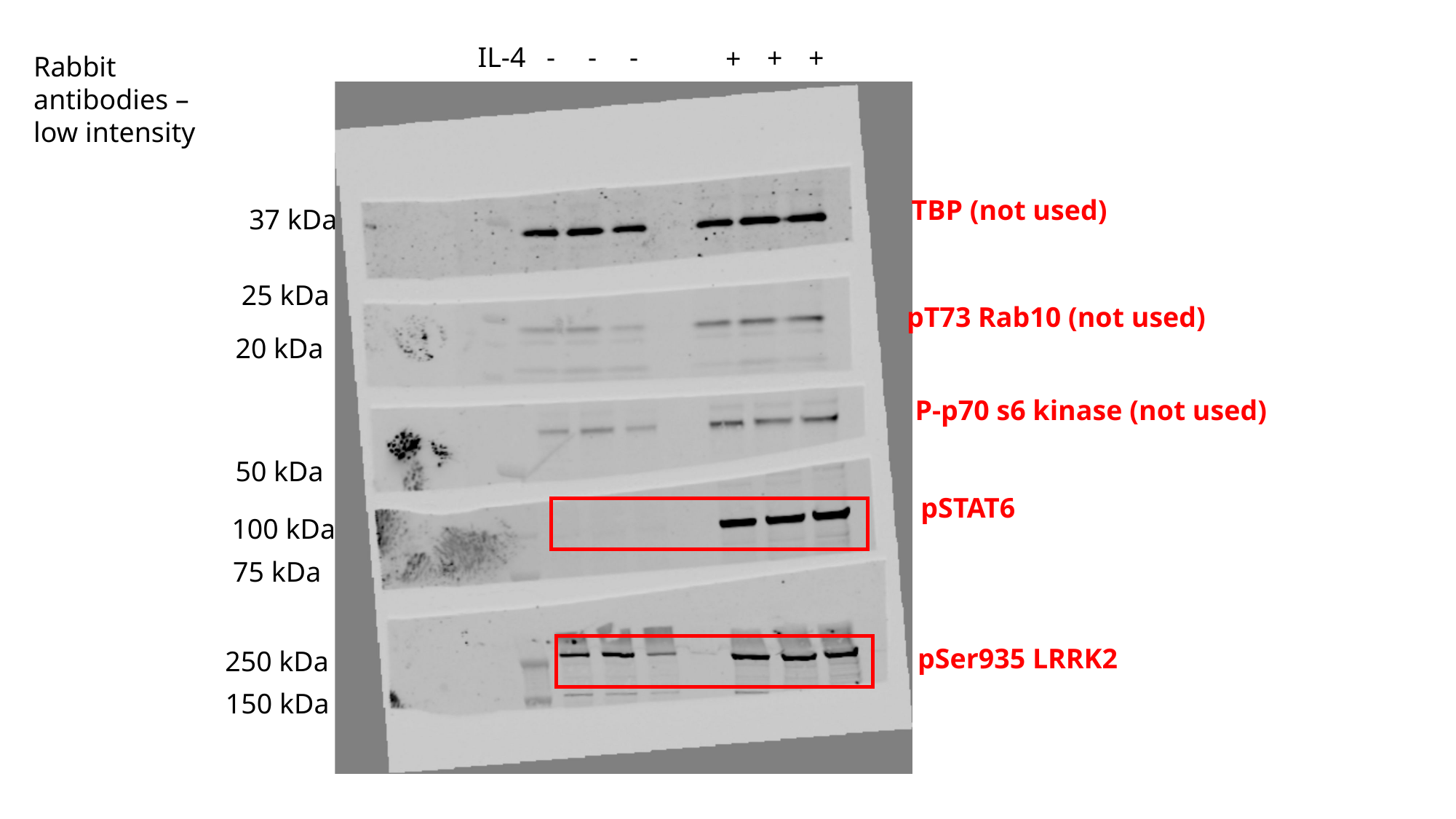

-
-
IL-4
-
+
+
+
Rabbit antibodies – low intensity
TBP (not used)
37 kDa
25 kDa
pT73 Rab10 (not used)
20 kDa
P-p70 s6 kinase (not used)
50 kDa
pSTAT6
100 kDa
75 kDa
pSer935 LRRK2
250 kDa
150 kDa

## Slide 3
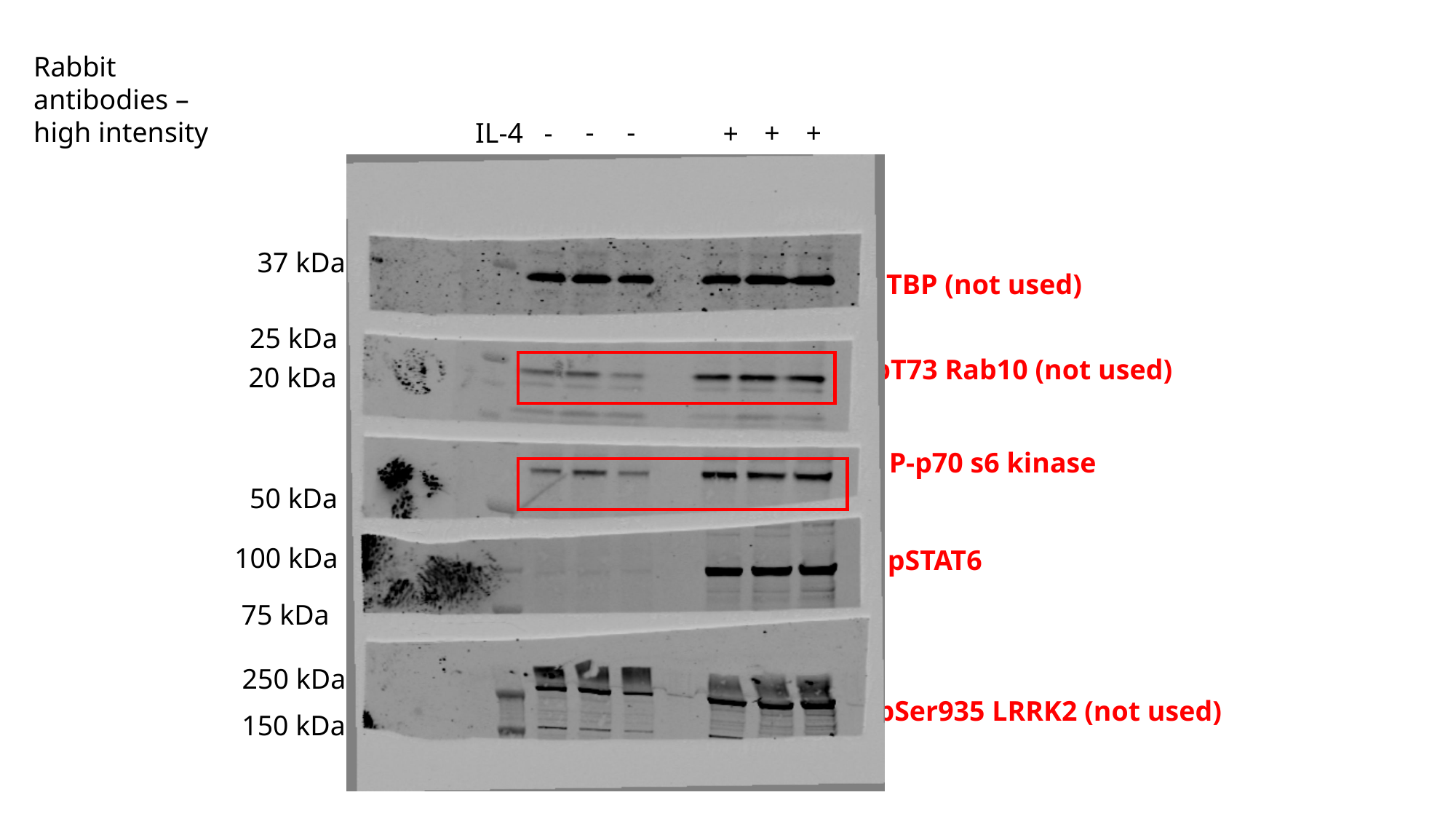

Rabbit antibodies – high intensity
-
-
IL-4
-
+
+
+
37 kDa
TBP (not used)
25 kDa
pT73 Rab10 (not used)
20 kDa
P-p70 s6 kinase
50 kDa
100 kDa
pSTAT6
75 kDa
250 kDa
pSer935 LRRK2 (not used)
150 kDa

## Slide 4
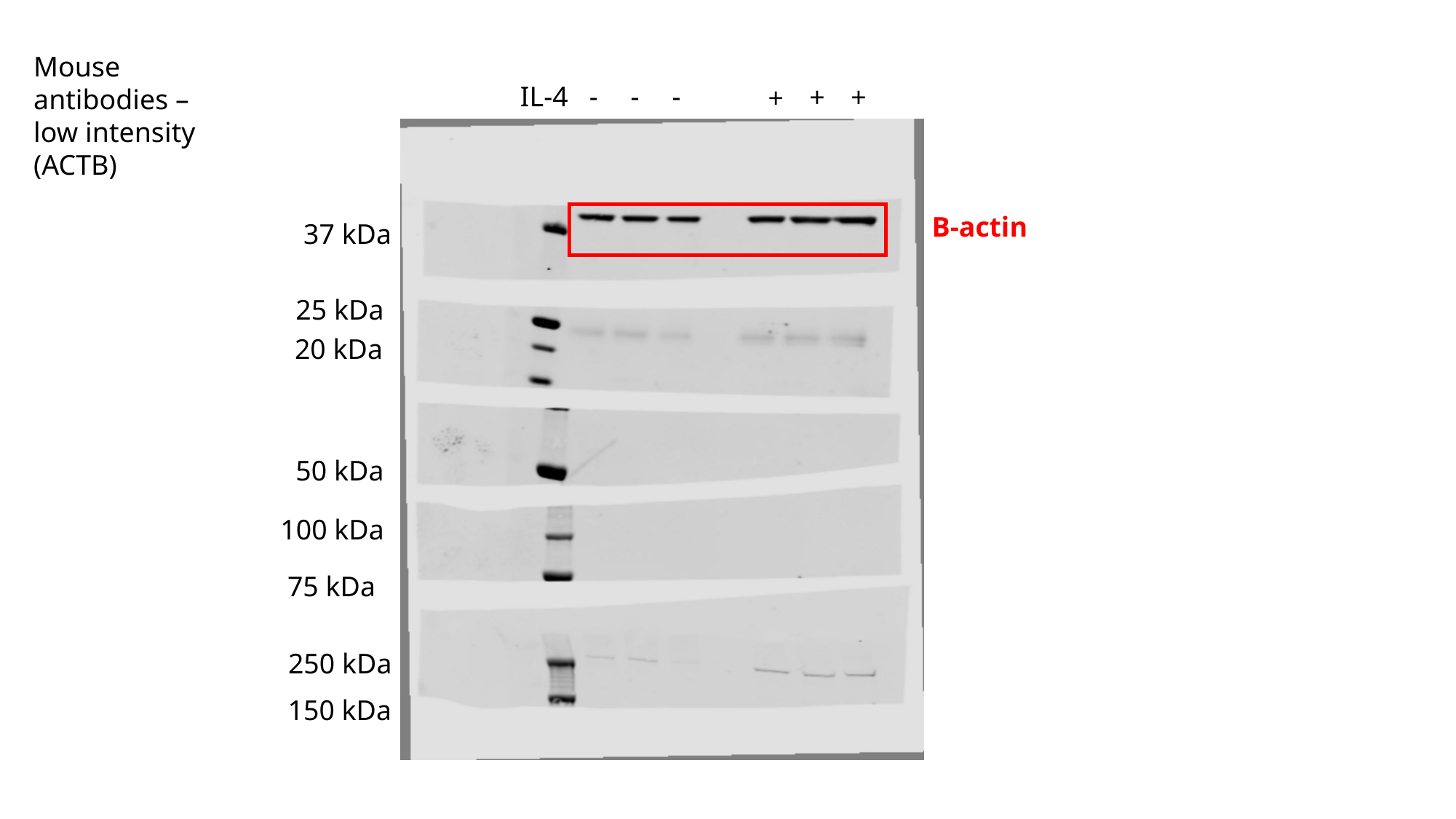

Mouse antibodies – low intensity (ACTB)
-
-
IL-4
-
+
+
+
B-actin
37 kDa
25 kDa
20 kDa
50 kDa
100 kDa
75 kDa
250 kDa
150 kDa

## Slide 5
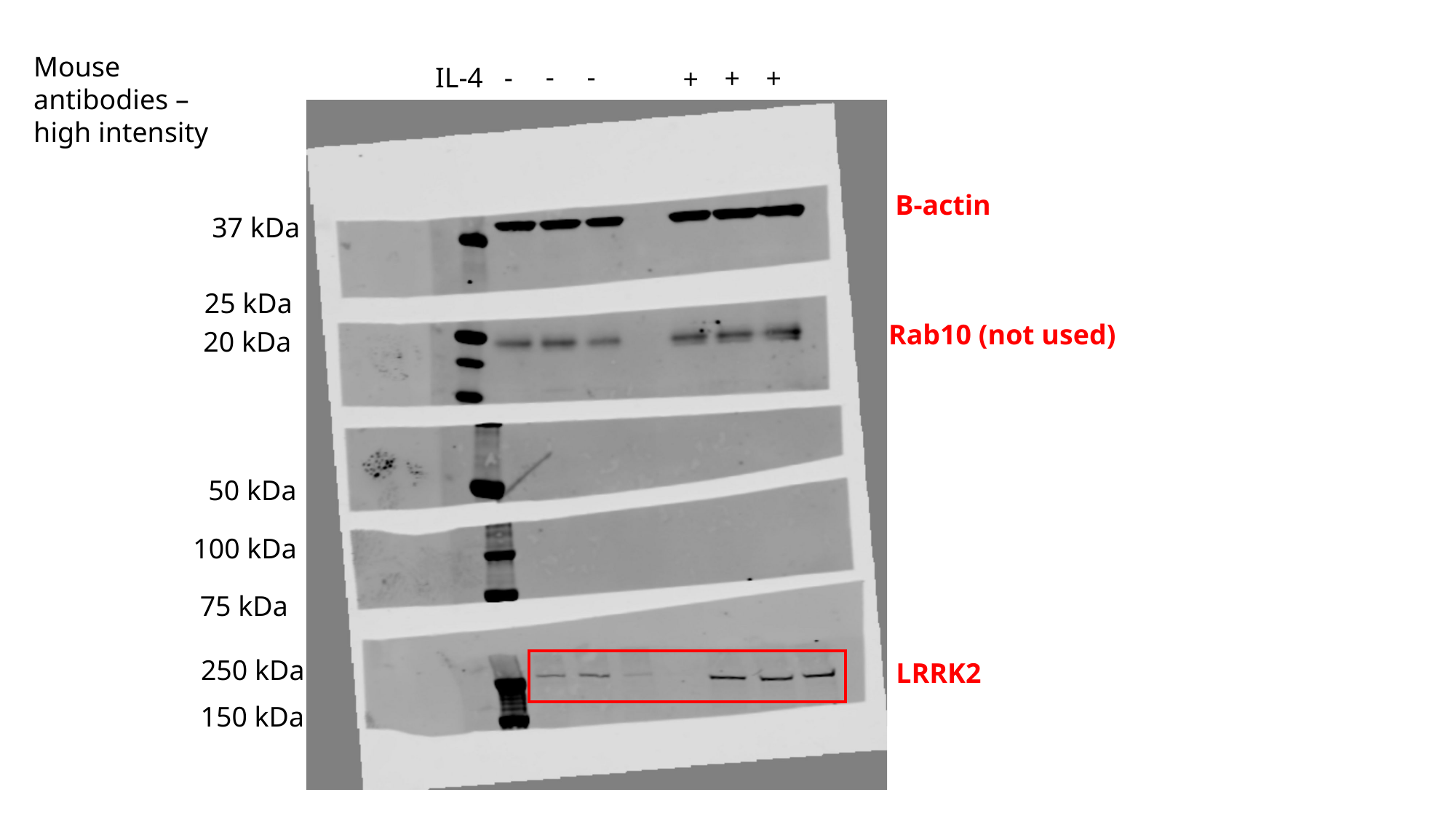

Mouse antibodies – high intensity
-
-
IL-4
-
+
+
+
B-actin
37 kDa
25 kDa
Rab10 (not used)
20 kDa
50 kDa
100 kDa
75 kDa
250 kDa
LRRK2
150 kDa

## Slide 6
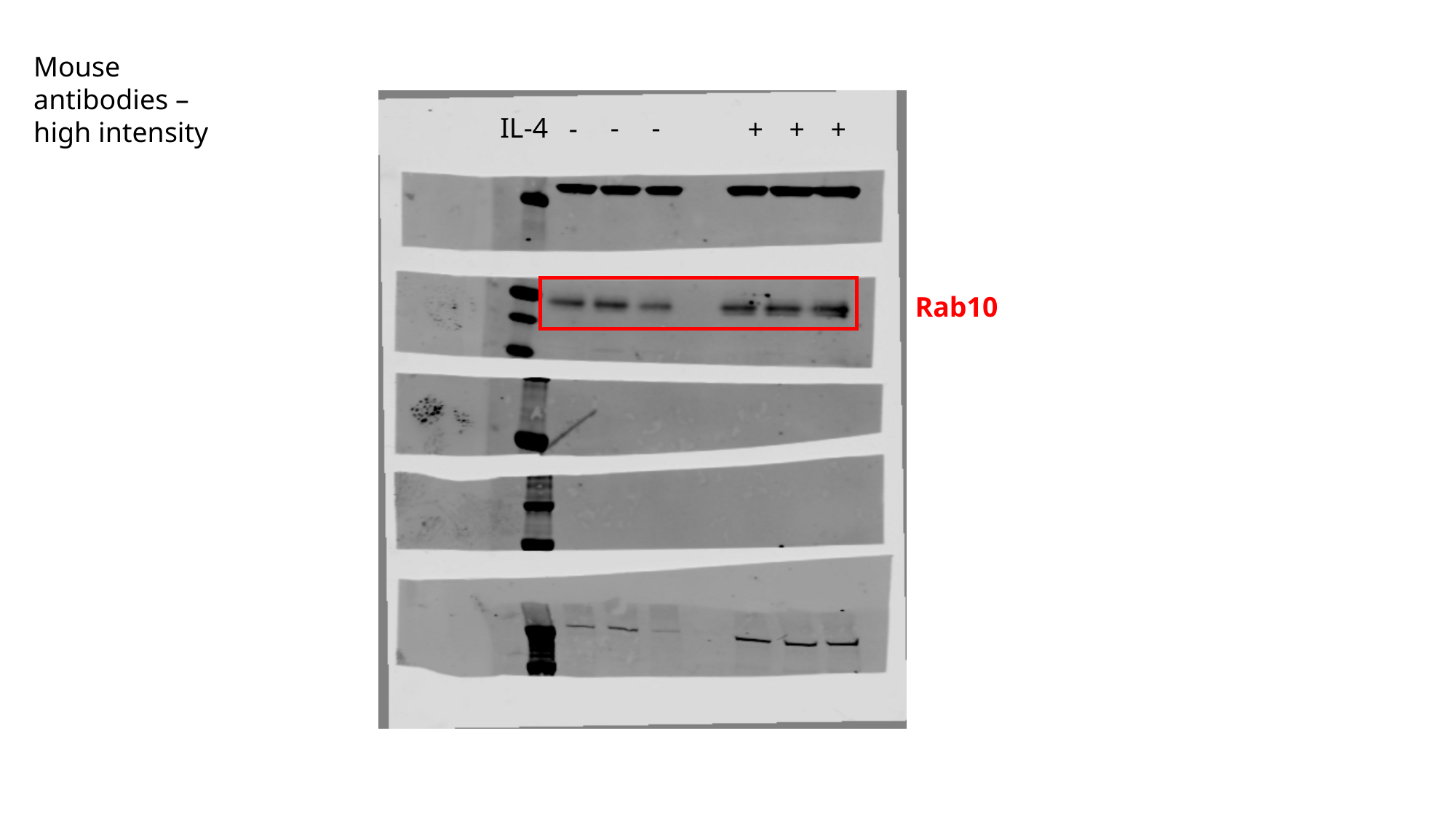

Mouse antibodies – high intensity
-
-
IL-4
-
+
+
+
Rab10
