# Supplementary figures and images for: Inflammation and IL-4 regulate Parkinson’s and Crohn’s disease associated kinase LRRK2
Source: EMBO Rep. 2025 May 20;26(13):3327–56. doi: 10.1038/s44319-025-00473-x (PMC12238514; doi:10.1038/s44319-025-00473-x)

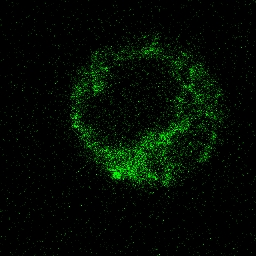

Supplement: Supplementary file 7 — Source data Fig. 5 [file 44319_2025_473_MOESM7_ESM.zip › Figure 5/5H/0_EGFP-LRRK2_B2cntr1.tiff]

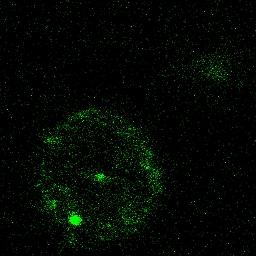

Supplement: Supplementary file 7 — Source data Fig. 5 [file 44319_2025_473_MOESM7_ESM.zip › Figure 5/5H/1_EGFP-LRRK2_B2cntr2.tiff]

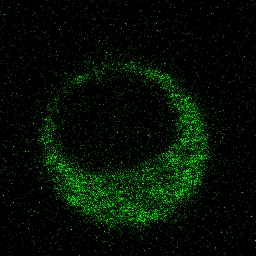

Supplement: Supplementary file 7 — Source data Fig. 5 [file 44319_2025_473_MOESM7_ESM.zip › Figure 5/5H/2_EGFP-LRRK2_B2_IL4_1.tiff]

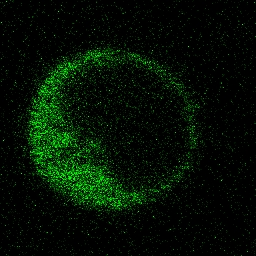

Supplement: Supplementary file 7 — Source data Fig. 5 [file 44319_2025_473_MOESM7_ESM.zip › Figure 5/5H/3_EGFP-LRRK2_B2_IL4_2.tiff]

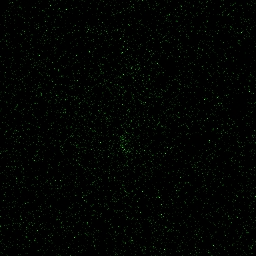

Supplement: Supplementary file 7 — Source data Fig. 5 [file 44319_2025_473_MOESM7_ESM.zip › Figure 5/5H/4_WT_B1cntr.tiff]

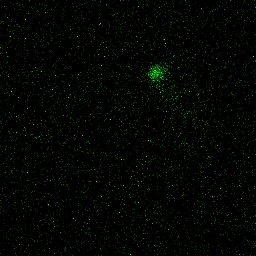

Supplement: Supplementary file 7 — Source data Fig. 5 [file 44319_2025_473_MOESM7_ESM.zip › Figure 5/5H/5_WT_B1_IL4.tiff]

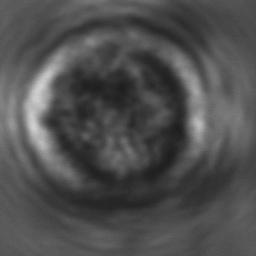

Supplement: Supplementary file 7 — Source data Fig. 5 [file 44319_2025_473_MOESM7_ESM.zip › Figure 5/5H/6_WT_T-PMT_B1cntr.tiff]

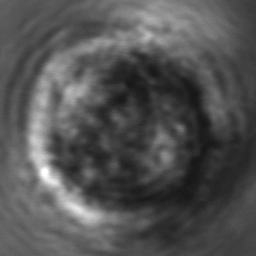

Supplement: Supplementary file 7 — Source data Fig. 5 [file 44319_2025_473_MOESM7_ESM.zip › Figure 5/5H/7_WT_T-PMT_B1_IL4.tiff]
