# Supplementary material for: Inflammation and IL-4 regulate Parkinson’s and Crohn’s disease associated kinase LRRK2
Source: EMBO Rep. 2025 May 20;26(13):3327–56. doi: 10.1038/s44319-025-00473-x (PMC12238514; doi:10.1038/s44319-025-00473-x)
Supplement: Supplementary file 8 — Expanded View Figures [file 44319_2025_473_MOESM8_ESM.pdf]

Expanded View Figures

Figure EV1. Immunofluorescence of mouse tissues with c41-2 anti-LRRK2 antibodies.

(**A**) Sections from paraffin-embedded ileum from a C57Bl/6 J (WT, rows 1 and 3) or a *Lrrk2*$^{-/-}$ mouse (rows 2 and 4) were co-stained with rabbit c41-2 anti-LRRK2 ab (clone MJFF2), red, and mouse anti-E-cadherin ab (green), followed by secondary anti-rabbit Alexa Fluor-568 (AF568) and anti-mouse Alexa Fluor-488 (AF488) antibodies, counterstained with DAPI (blue) and imaged by confocal microscopy (panels 1 and 2 from left). Staining that did not include primary antibodies used as a control (panels 3 and 4) was processed, imaged and adjusted in in the same way. Single optical sections are shown. Panels 2 and 4 depict areas with Peyer's Patches. Top two rows show overlay of all staining, and the bottom two rows show only c41-2 channel from the same images in black and white. Scale bar = 100 µm. Representative of at least four similar experiments. (**B**) Sections from frozen lung tissues from WT (top) or *Lrrk2*$^{-/-}$ (bottom) mice were stained with c41-2 anti-LRRK2 ab (clone MJFF2) (red on overlay) followed with Alexa Fluor-568 fluorescent secondary ab (panels 1 and 2), or with secondary ab only (panels 3 and 4). Sections were counterstained by DAPI (blue) and Phalloidin-488 (not shown). Confocal images were acquired and processed identically. Panels 1 and 3 show c41-2 and DAPI overlay, panels 2 and 4 show c42-1 channel only in black and white. Scale bar = 50 µm. Images were processed and assembled in OMERO.

© The Author(s)

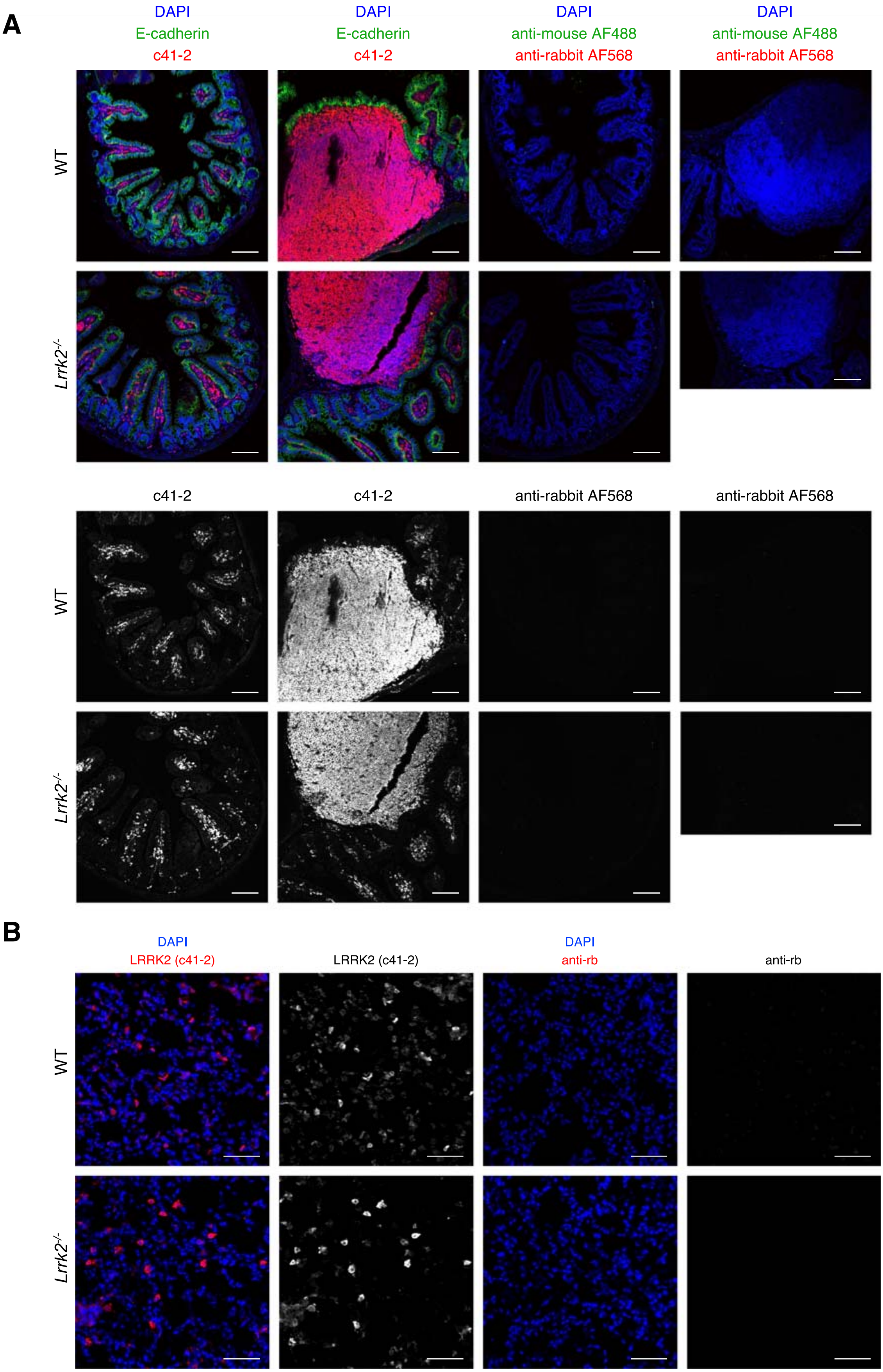
A
DAPI
E-cadherin
c41-2
DAPI
E-cadherin
c41-2
DAPI
anti-mouse AF488
anti-rabbit AF568
DAPI
anti-mouse AF488
anti-rabbit AF568
WT
Lrrk2-/-
c41-2
c41-2
anti-rabbit AF568
anti-rabbit AF568
WT
Lrrk2-/-
B
DAPI
LRRK2 (c41-2)
LRRK2 (c41-2)
DAPI
anti-rb
anti-rb
WT
Lrrk2-/-

 © The Author(s)

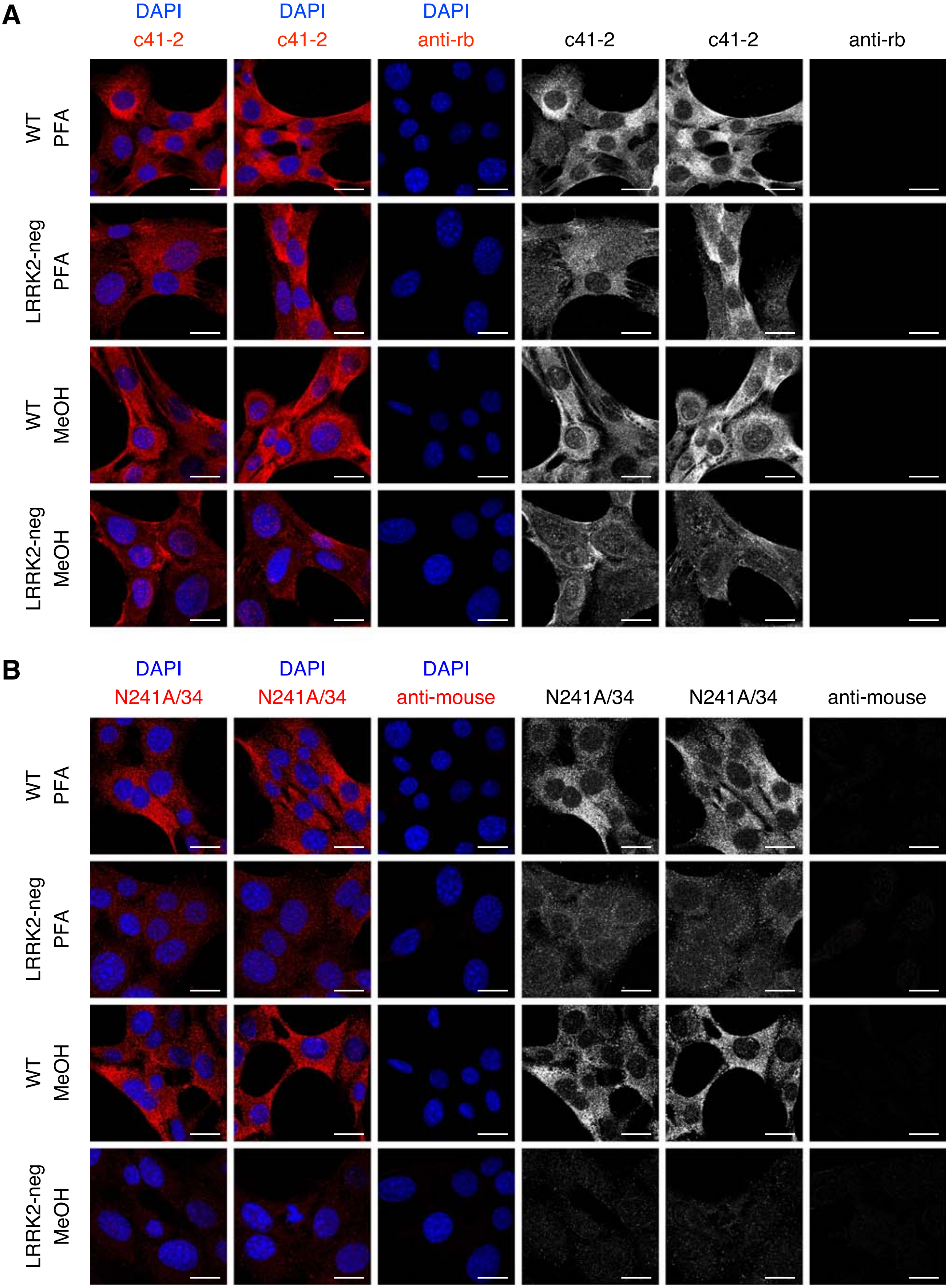
A
DAPI
c41-2
DAPI
c41-2
DAPI
anti-rb
c41-2
c41-2
anti-rb
WT PFA
LRRK2-neg PFA
WT MeOH
LRRK2-neg MeOH
B
DAPI
N241A/34
DAPI
N241A/34
DAPI
anti-mouse
N241A/34
N241A/34
anti-mouse
WT PFA
LRRK2-neg PFA
WT MeOH
LRRK2-neg MeOH

© The Author(s)

Figure EV2. LRRK2 immunofluorescence in mouse MODE-K cell line.

Parental MODE-K cells (WT) and the LRRK2-deficient clone (LRRK2-neg) generated by CRISPR-Cas9 knock-out of LRRK2 were grown on coverslips, fixed/permeabilised in either 4% PFA / 1% Triton X-100 (top two rows, PFA) or Methanol (bottom two rows, MeOH), and stained with either c41-2 (clone MJFF2) (**A**) or clone N241A/34 (**B**) anti-LRRK2 antibodies followed by an appropriate fluorescently-labelled secondary ab (red on overlay) and DAPI (blue on overlay). Cells were imaged on confocal microscope and images processed in OMERO. Anti-rabbit (anti-rb) or anti-mouse secondary-only controls (panels 3 and 6) were imaged and processed identically to the full-stained samples. The overlays are shown in panels 1-3, and panels 4-6 depict LRRK2-only staining in black and white. Scale bar = 20 µm.

 © The Author(s)

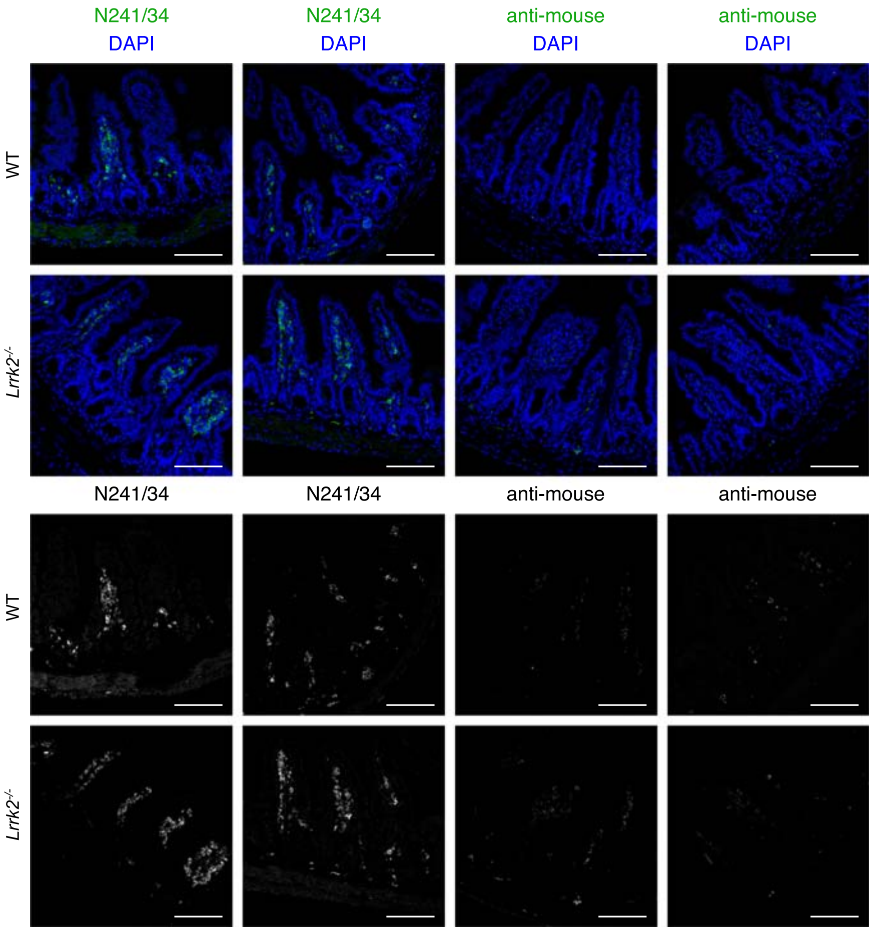

Figure EV3. Tissue immunofluorescence with anti-LRRK2 antibody N241A/34.

Sections of paraffin-embedded ileums from a *Lrrk2*$^{-/-}$ mouse (rows 2 and 4) and its WT littermate (rows 1 and 3) were stained with N241A/34 antibodies followed by fluorescent secondary ab (panels 1 and 2) or with secondary ab only (panels 3 and 4), (green on overlay) and counterstained with DAPI (blue on overlay). Tissues were imaged on confocal microscope and processed in OMERO. Top two rows show overlay, bottom two rows show LRRK2-only staining in black and white. Scale bar = 100 µm.

© The Author(s)

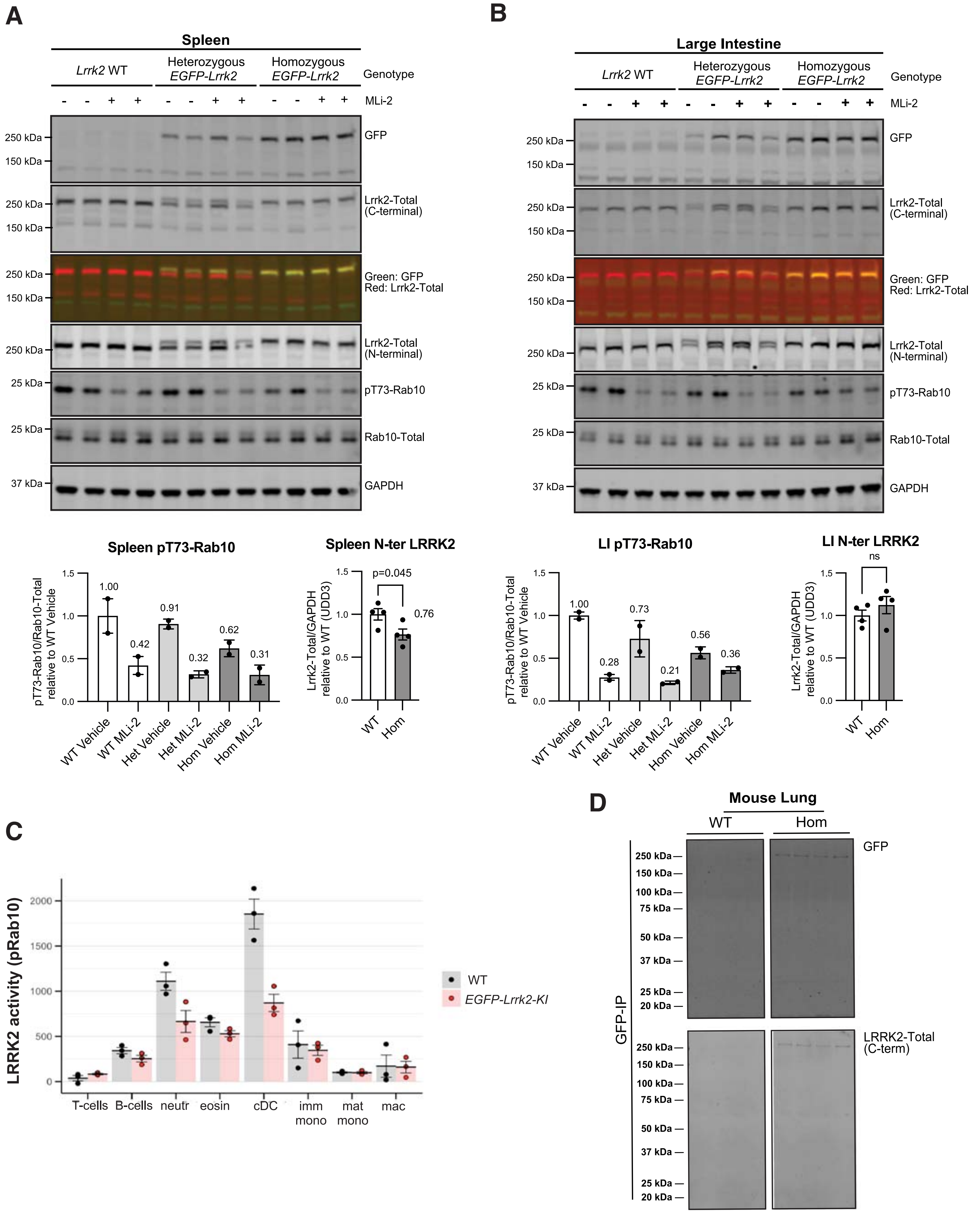
A
Spleen
Lrrk2 WT
Heterozygous EGFP-Lrrk2
Homozygous EGFP-Lrrk2
Genotype
MLi-2
GFP
Lrrk2-Total (C-terminal)
Green: GFP
Red: Lrrk2-Total
Lrrk2-Total (N-terminal)
pT73-Rab10
Rab10-Total
GAPDH
Spleen pT73-Rab10
Spleen N-ter LRRK2
p=0.045
B
Large Intestine
LI pT73-Rab10
LI N-ter LRRK2
ns
WT Vehicle
WT MLi-2
Het Vehicle
Het MLi-2
Hom Vehicle
Hom MLi-2
C
LRRK2 activity (pRab10)
WT
EGFP-Lrrk2-KI
T-cells
B-cells
neutr
eosin
cDC
imm mono
mat mono
mac
D
Mouse Lung
WT
Hom
GFP-IP
GFP
LRRK2-Total (C-term)

© The Author(s)

Figure EV4. Analysis of the LRRK2 pathway in tissues from *EGFP-Lrrk2-KI* mouse model.

(**A**, **B**) 3-month-old WT, heterozygous and homozygous *EGFP-Lrrk2-KI* mice ($n = 2$ each) were treated with or without 30 mg/kg MLi-2 subcutaneously for 2 h prior to culling. Mouse tissues were immediately extracted and snap frozen in liquid nitrogen. Frozen tissues were lysed using Cryolys Evolution and between 20 to 30 µg of whole tissue lysate of either spleen (**A**) or large intestine (**B**) subjected to immunoblot analysis. Quantification of LRRK2-substrate phosphorylation of pT73 Rab10 relative to total levels ($n = 2$), and total LRRK2 levels relative to GAPDH for N-terminal antibodies ($n = 4$) are shown as mean ± SEM for each tissue. Each lane indicated sample derived from a different mouse tissue. Statistical significance was assessed by two-tailed unpaired *T* test. ns = not significant. (**C**) LRRK2 activity (pRab10) was measured in splenocytes from homozygous *EGFP-Lrrk2-KI* mice (EGFP-LRRK2-KI, red, $n = 3$) or their wild-type littermates (WT, black, $n = 3$) and displayed as in Fig. 1D. Dots show measurements in individual mice, bars with error bars show means ± SEM. (**D**) Mouse lung tissues derived from homozygous genotypes of Lrrk2-WT ($n = 4$) and *EGFP-Lrrk2-KI* ($n = 4$) mice were homogenised using Cryolys Evolution in 0.5% NP-40 detergent lysis buffer. 4 mg of lung tissue lysate was subjected to a GFP-IP at 4 °C for 2 h. 10% of the IP eluate was subjected to immunoblot analysis using antibodies indicated.

© The Author(s)

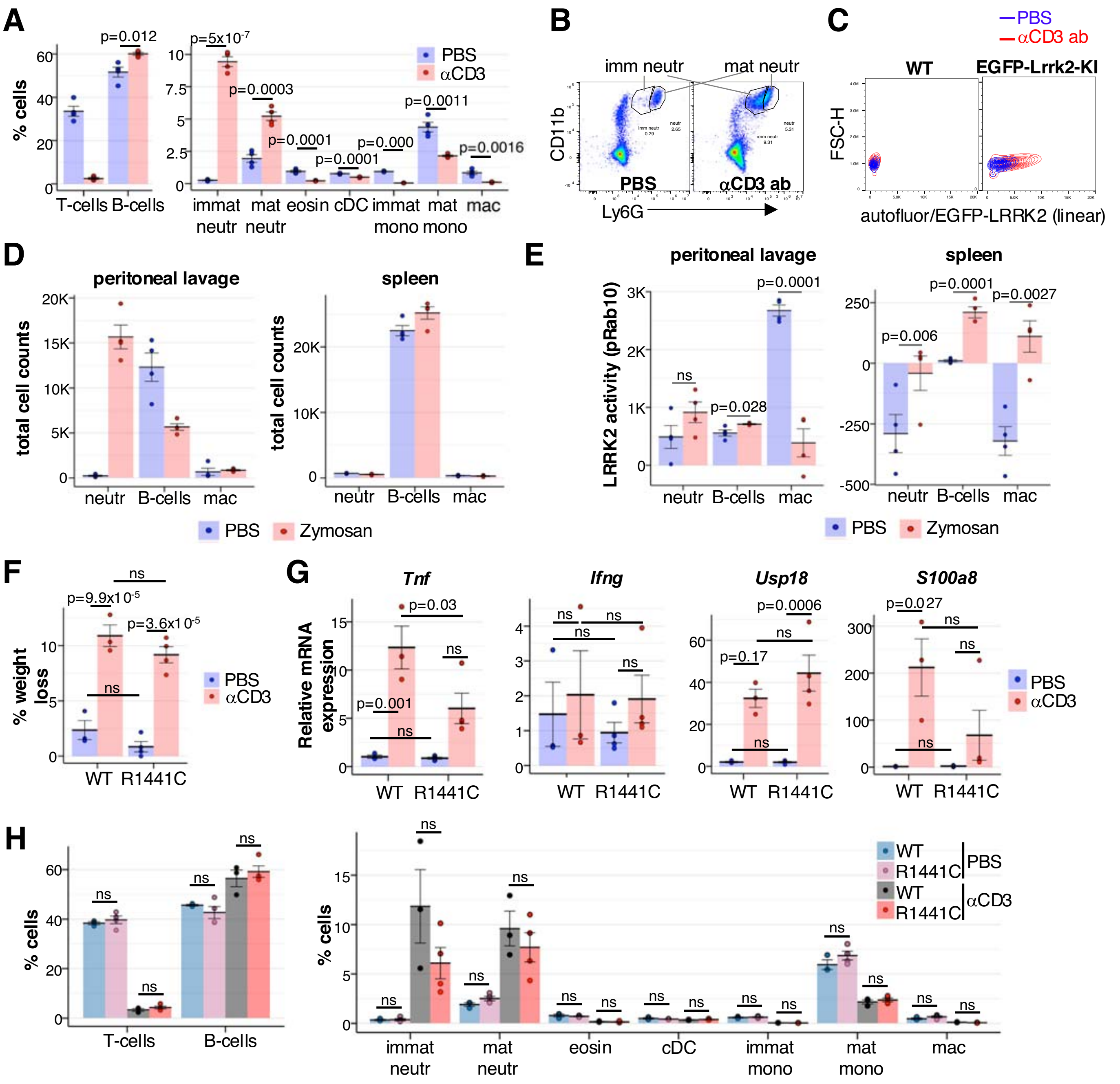
A
% cells
T-cells B-cells
immat neutr
mat neutr
eosin
cDC
immat mono
mat mono
mac
PBS
αCD3
B
imm neutr
mat neutr
CD11b
Ly6G
PBS
αCD3 ab
C
PBS
αCD3 ab
WT
EGFP-Lrrk2-KI
FSC-H
autofluor/EGFP-LRRK2 (linear)
D
peritoneal lavage
spleen
total cell counts
neutr B-cells mac
PBS Zymosan
E
peritoneal lavage
spleen
LRRK2 activity (pRab10)
neutr B-cells mac
PBS Zymosan
F
% weight loss
WT R1441C
PBS
αCD3
G
Tnf
Ifng
Usp18
S100a8
Relative mRNA expression
WT R1441C
PBS
αCD3
H
% cells
T-cells
B-cells
WT
R1441C
PBS
WT
R1441C
αCD3
immat neutr
mat neutr
eosin
cDC
immat mono
mat mono
mac

 © The Author(s)

Figure EV5. Effects of inflammation on LRRK2 activity in WT and Lrrk2-R1441C mutant mice.

(**A**) Cellular composition of mouse spleen ($n = 4$ mice) 24 h after i.p. injection of anti-CD3 (αCD3, red) or PBS (blue). Frequencies of indicated cell types as a percent of single live CD45^{+} cells is shown. Statistical analysis was not applied to T cells, since strong reduction in the number of T cells is most likely due to incomplete identification of T cells resulting from anti-CD3 induced internalisation of the TCR. (**B**) Distinction between mature and immature neutrophils by Ly6G level from cells in A. Note a strong expansion of the CD11b^{hi}/Ly6G^{int} immature neutrophil subset in anti-CD3 ab injected sample. (**C**) EGFP-LRRK2 fluorescence (for *EGFP-Lrrk2-KI* sample) or autofluorescence measured in the same channel (for matching WT control) in B cells among splenocytes from WT (left panel) or EGFP-Lrrk2-KI (right panel) mice 24 h after anti-CD3 (red) or PBS (blue) injection, plotted on a linear scale against FSC-H. (**D**, **E**) WT mice were i.p. injected with either 1 mg/mouse Zymosan A (red, $n = 4$) or vehicle (PBS, blue, $n = 4$). 18 h later the total numbers of neutrophils, B cells and macrophages (**D**) and their LRRK2 activity (**E**) were measured in peritoneal lavage (left) and spleens (right) and displayed as in A. (**F**). Body weights before and 24 h after i.p. injection of anti-CD3 antibody (αCD3, red, $n = 3$ and 4) or equal volume of PBS (PBS, blue, $n = 3$ and 4) were measured for wild-type (WT) and *Lrrk2-R1441C* knock-in (R1441C) mice, respectively, and % weight loss was calculated. (**G**) Relative expression of *Tnf*, *Ifng*, *Usp18* and *S100a8* mRNA was measured by qPCR in ileums isolated from *Lrrk2-R1441C* mice (R1441C) and their wild-type littermates (WT) 24 h after anti-CD3 (red, $n = 4$ and 3) or PBS (blue, $n = 4$ and 3) injection. Graphs show fold changes relative to PBS-injected WT mice, with *Tbp* as a reference gene. (**H**). Frequencies of indicated cell types among live single CD45+ splenocytes obtained from WT or R1441C mice 24 h after anti-CD3 (grey, $n = 3$, or red, $n = 4$) or PBS (purple, $n = 3$, or blue, $n = 4$) injection are displayed. Note that there was no statistically significant difference between two genotypes. Data information: (**A**, **E**, **D**–**H**): Dots depict values in individual mice, bars show means, and error bars represent SEM. Statistical significance calculated by one-way ANOVA (**A**), 2-way ANOVA (**F**–**H**) or 2-tailed T test (**E**) is shown as P values or ns = not significant.

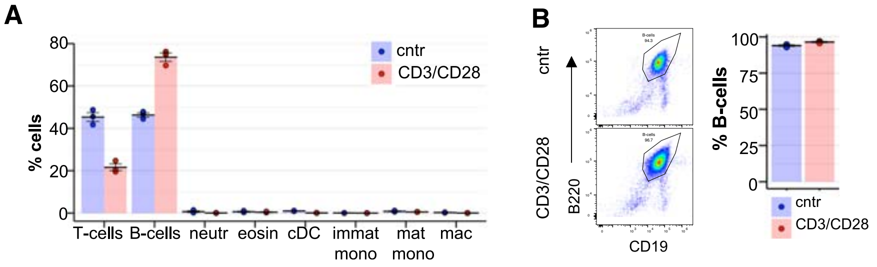

Figure EV6. In vitro B-cell stimulation by T-cell-dependent factors.

(**A**) Frequencies of indicated cell types in total splenocyte cultures 24 h after in vitro incubation with immobilised anti-CD3/CD28 antibodies (CD3/CD28, red, $n = 3$) or in control media supplemented with IL-7 (cntr, blue, $n = 3$) were quantified as % from alive single CD45+ cells within harvested cell suspensions. (**B**) Purity of B cells isolated from mixed splenocyte cultures after 24 h incubation with immobilised anti-CD3/CD28 antibodies (CD3/CD28, bottom left panel, red on the plot) or in control media supplemented with IL-7 (cntr, top left panel, blue on plot). B cells were purified using negative selection kit and defined as CD19+/B220+ cells (left panels). The quantification of the purity as % from single live cells is shown on the right (3 per group). Data information: (**A**, **B**) Values from individual mice (dots), means (bars) and SEM (error bars) are shown.

 © The Author(s)
